# The Analysis of the Urea Biosensors Using Different Sensing Matrices via Wireless Measurement System & Microfluidic Measurement System

**DOI:** 10.3390/s19133004

**Published:** 2019-07-08

**Authors:** Jung-Chuan Chou, Cian-Yi Wu, Si-Hong Lin, Po-Yu Kuo, Chih-Hsien Lai, Yu-Hsun Nien, You-Xiang Wu, Tsu-Yang Lai

**Affiliations:** 1 Graduate School of Electronic Engineering, National Yunlin University of Science and Technology, Douliu 64002, Taiwan; 2 Graduate School of Chemical and Materials Engineering, National Yunlin University of Science and Technology, Douliu 64002, Taiwan

**Keywords:** nickel oxide (NiO), titanium dioxide (TiO_2_), urea, microfluidics, wireless detection

## Abstract

Two types of urea biosensors were integrated with a wireless measurement system and microfluidic measurement system. The two biosensors used were (i) a magnetic beads (MBs)-urease/graphene oxide (GO)/titanium dioxide (TiO_2_)-based biosensor and (ii) an MBs-urease/GO/ nickel oxide (NiO)-based biosensor, respectively. The wireless measurement system work exhibited the feasibility for the remote detection of urea, but it will require refinement and modification to improve stability and precision. The microchannel fluidic system showed the measurement reliability. The sensing properties of urea biosensors at different flow rates were investigated. From the measurement results, the decay of average sensitivity may be attributed to the induced vortex-induced vibrations (VIV) at the high flow rate. In the aspect of wireless monitoring, the average sensitivity of the urea biosensor based on MBs-urease/GO/NiO was 4.780 mV/(mg/dl) and with the linearity of 0.938. In the aspect of measurement under dynamic conditions, the average sensitivity of the urea biosensor based on MBs-urease/GO/NiO were 5.582 mV/(mg/dl) and with the linearity of 0.959. Both measurements performed NiO was better than TiO_2_ according to the comparisons.

## 1. Introduction

In the human body, consumed protein forms amino acids after hydrolysis in the digestive system. Subsequently, the urea cycle in the liver is where the main metabolic process occurs [1]. Urea that directly reaches the blood stream from the kidneys is also egested in urine. The normal urea concentration range in the human body is from 2.5 mM to 7.5 mM. When the concentration of urea is abnormal, kidney disease and hyperammonemia may occur. A high urea concentration may lead to diseases including renal dysfunction, urinary tract obstruction, dehydration, shock, and gastrointestinal bleeding [2].

Two kinds of promising materials, nickel oxide (NiO) and titanium oxide (TiO_2_), have been developed for use as matrices in electronic urea biosensors, due to their unique chemical reactant properties. NiO advantages include chemical stability and a fast electron transfer capability [3,4,5,6]. Furthermore, it has been used as a sensing material for detection of glucose, urea, and uric acid, thus it has already established itself in the field of biomonitoring systems. TiO_2_ has the advantage that it is biocompatible, non-toxic, and has an electron transfer capability. Due to the biocompatibility of TiO_2_, there is an expected and appropriate reaction between TiO_2_ and the analyte. Coordination bonds can be formed by titanium, connecting with functional groups of enzymes. Additionally, TiO_2_ offers many outstanding properties, it is non-toxic, non-corrosive, environmentally-friendly, cost-effective, and has better electron transition. Additionally, it can also help maintain the biocatalytic activity of enzymes [7]. TiO_2_ showed significant promise in surface tests for the immobilization of biomolecules, therefore, recently increased research has been devoted to it [8,9,10,11,12,13]. The above researches all demonstrate that NiO and TiO_2_ are promising materials when they are used as sensing matrices. Besides, it is rare that urea sensors are exploited by using NiO and TiO_2_, and so we used them as the sensing matrices of the urea biosensors.

Graphene oxide (GO) and magnetic beads (MBs) have been used to modify the characteristics of urea biosensors. GO has abundant oxygen-containing functional groups, owing to the chemical process of Hummers’ method, wherein part of the carbon atoms is oxidized and the original carbon structure is destroyed. As numerous functional groups can be grafted onto the graphene layer during the preparation, it has abundant functional groups afterward for the reaction. In this paper, the use of graphene oxide between the enzyme layer and the magnetic beads layer is expected to adsorb and consolidate strongly, this expectation is based on the presence of abundant functional groups with enhanced electron transfer properties. Additionally, MBs provide other advantages, such as a wide range of biomagnetic separations, molecular manipulations, and affinity isolations. The ferroso ferric oxide (Fe_3_O_4_) and iron(III) oxide (Fe_2_O_3_) particles constitute MBs, which is closely related to polystyrene. Moreover, the beads were further coated in glycidyl ether, to create a hydrophilic layer that conceals the iron oxide. Carboxylic groups were also deposited onto the surface of the beads. In this study, the abundant oxygen-containing functional groups of GO and the high surface-volume ratio of MBs serve more sites for TiO_2_ or NiO, thereby enhancing sensing characteristics.

Over the past few years, interest in wearable devices for biophysical monitoring has been growing rapidly. Thus, a flexible and durable substrate, such as the one proposed here consisting of polyethylene terephthalate (PET), polyimide (PI), and polyethylene naphthalate (PEN) is a prime area for development that can be broadly applied in the market for wearable medical devices [14]. Screen-printing technology is the chance that electrodes are minimized on a PET substrate. In addition, these advantages of the applicability of cheap and mass-producible lead to sensitive, rapid, miniaturization, and reproducible biosensors, so much so that it is now an accepted way in the industry.

Monitoring in a long distance is indispensable along with the development of point-of-care. The ZigBee communication layer has many features that make it ideal for the task, such as low power consumption, low price, and simple construction that allows for modifications and development to enhance security. In addition, it also has highly reliable data management [15,16,17,18]. The ZigBee data standard is based on the standard IEEE 802.15.4 network protocol specifications so it is highly cross-compatible [17]. Apart from this, the researches on microfluidic measurement systems over the past few decades, a number of issues have arisen, and some of which remain controversial in spite of the vast data available [19,20,21]. Microfluidic systems have been widely used to integrate biosensors, which could offer advantages such as enhanced sensitivity, the mixing rate of different reagents, conditions of flow control, abate reagents volume, and the ability to unite chemical and biological components into a single platform [22,23].

**Figure 1:**
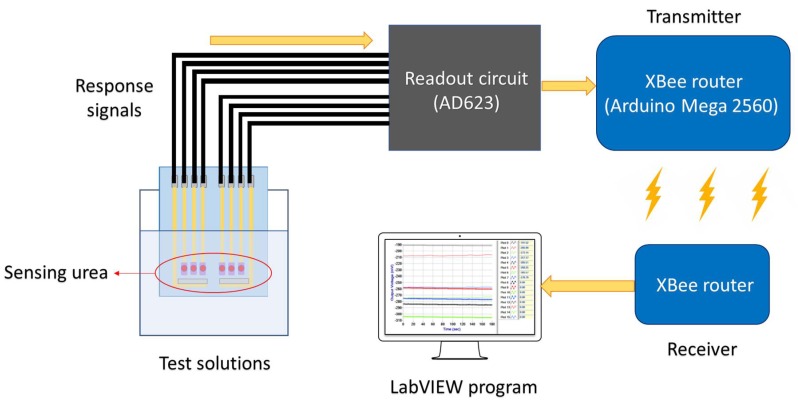
The schematic of the wireless measurement system, which includes a urea biosensor, a readout circuit, an XBee module, and a computer with the LabVIEW program.

Very recently, it is rare that the reports about what the remote detection of urea and the application on microfluidics were investigated simultaneously. Apart from this, TiO_2_ or NiO are exceptional sensing matrices that can promote the conduction of interfacial electrons and are beneficial for binding the enzyme [24]. Therefore, this paper inherits the previous researches [25,26] and further explores the sensing characteristics, which the urea biosensors are integrated with the wireless measurement system and the microfluidic measurement system. Microfluidic systems are commonly used to analyze the concentration of the specific substance continuously, owing to the fast response time, the small uses of amounts of reagents, and automation [22,23]. In order to accomplish continuous analysis and point-of-care diagnostics for urea, we measured and analyze the dynamic sensing characteristics of the urea biosensor under microfluidic flow characteristics. In terms of the remote detection of urea, the urea biosensors were integrated with the wireless sensing system based on ZigBee standard, the concentration was detected and shown on the screen at once to realize real-time diagnostics. Figure 1 shows the wireless measurement system, which includes a urea biosensor, a readout circuit, an XBee module, and a computer with the LabVIEW program.

## 2. Materials and Methods

### 2.1. Materials

Nickel oxide (NiO) with a target of 99.95% purity, purchased from Ultimate Materials Technology Co., Ltd. (Hsinchu County, Taiwan), was used to produce the sensing film. The titanium dioxide (TiO_2_) with a target of 99.95% purity was purchased from Ultimate Materials Technology Co., Ltd. (Hsinchu County, Taiwan), this was used for deposition on the sensing film. The graphite powder was purchased from Alfa Aesar Co. (Lancashire, UK), was used to synthesize the graphene oxide (GO) by the modified Hummers’ method [27]. Magnetic beads (MBs) were purchased from Quantum Biotechnology Inc. (Taichung City, Taiwan), belong to Dynabeads, have non-toxic nature in cell-based clinical applications [28], were used to enhance the enzymatic immobilization. N-Ethyl-N’- (3-dimethylaminopropyl) carbodiimide hydrochloride (EDC), was purchased from Sigma-Aldrich, Co. (St. Louis, MO, USA), and was used as a carboxyl activating agent to bind the MBs and enzyme. Phosphate monobasic (KH_2_PO_4_) powders and potassium phosphate dibasic (K_2_HPO_4_) powders were purchased from Katayama Chemical Industries Co., Ltd. (Yatsuo Machi, Japan), were used to prepare 30 mM phosphate buffer saline solutions (PBS) with pH 7 (this pH is a common value for human urine or human serum). Urease was purchased from Sigma-Aldrich Corp. (St. Louis, MO, USA). Urea powders were purchased from J. T. Baker Corp. (St. Louis, MO, USA). Polyethylene terephthalate (PET) was purchased from Zencatec Corporation (Tao-Yuan City, Taiwan). Silver paste was purchased from Advanced Electronic Material Inc. (Tainan City, Taiwan), and was used for the conductive wire and reference electrode. The epoxy thermosetting polymer (product no. JA643) was purchased from Sil-More Industrial, Ltd. (New Taipei City, Taiwan).

### 2.2. Fabrication of the Urea Biosensors Based on NiO and TiO_2_

The fabrication processes of the urea biosensor were as follows:

1. The polyethylene terephthalate (PET) substrate was cut into 30 mm × 40 mm. The PET was used as the substrate of the urea biosensors.
2. The pattern was used as the arrayed conductive wires and reference electrodes, which silver paste was screen-printed onto a PET flexible substrate and was baked in an oven at 120 °C. The silver paste exhibited excellent adhesion to a PET substrate, and excellent electrical conduction capabilities. The sensor array was preliminarily tested and found to be working normally after being tested.
3. TiO_2_ and NiO sensing films as matrices were prepared via using the radio frequency (R.F.) sputtering system. The sputtering parameters for the different sensing matrices are shown in Table 1.
4. The flexible arrayed urea biosensor was encapsulated with epoxy. The epoxy, which also served as an insulation layer, could be used to define as the sensing area per window of 1.77 mm^2^.

### 2.3. Preparation and Modification of the MBs-Urease Solution

1. The 0.3 wt% GO solution was prepared by mixing GO powder and D.I. water, it was then titrated onto the sensing matrices (TiO_2_ and NiO).
2. The N-Ethyl-N’- (3-dimethylaminopropyl) carbodiimide hydrochloride (EDC) solution and MBs were mixed evenly. The duration of the process is owing to the surface functional groups of MBs, which could make activation of carboxyl and hydroxyl for MBs. Then, the urease mixed and vibrated MBs for 8 h. The optimal volume ratio of MBs-urease is 1:1. The MBs-urease composite solution was dropped onto the sensing matrices (GO/TiO_2_ and GO/NiO).

**Figure 2:**
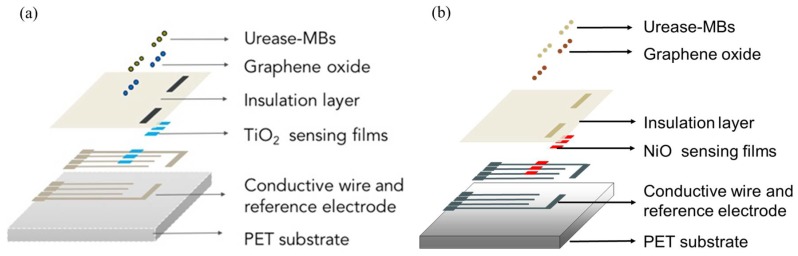
The structure schematics of (a) the MBs-urease/GO/TiO2-based biosensor [25] and (b) the MBs-urease/GO/NiO-based biosensor [26].

The detailed steps for the fabrication of the biosensors can be found in our previous study [25,26], and the structure schematics are shown in Figure 2.

In this study, urease is used as the enzyme electrode. Urea is catalyzed and hydrolyzed by urease, as depicted in the reaction Formula (1):

(1) $$NH_{2}CONH_{2}+3H_{2}O\overset{Urease}{\to }2NH_{4}^{＋}+HCO_{3}^{-}+OH^{-}$$

Urea is converted into ammonium ions (NH_4_^+^), bicarbonate ions (HCO_3_^−^), and hydroxide ions (OH^−^) by urease. The surface potential of the sensing electrode depends on the variation of produced OH^−^, resulting in the different potential difference between the sensing electrode and reference electrodes. Next, the signals were processed by the readout circuit and the computer to realize the detection of urea.

### 2.4. Wireless Measurement System

**Figure 3:**
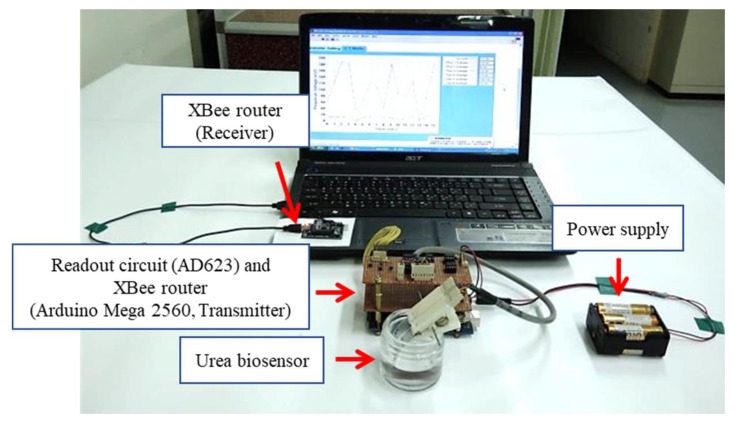
The photo of the wireless measurement system.

The device platform used here is shown in Figure 3 and combines an XBee module (Digi International, Hopkins, MN, USA) readout circuit (AD623), the Arduino Mega 2560, a microcontroller board based on the ATmega 2560 from Atmel Corporation (San Jose, CA, USA), and a power supply. The wireless measurement system is based on ZigBee standard to transmit the response signals from the biosensor. The input voltage to the device could be switched between 3.3 V and 5 V by the Arduino Mega 2560, which provided enough power that the XBee router and readout circuit could function correctly. The Arduino Mega 2560 was used for conversion of the analog signal to a digital signal. The XBee router (which includes the Arduino Mega 2560) was used for receiving the signals from the sensor and transmitting them to the XBee coordinator. Lastly, the signals were converted in the computer for use with the LabVIEW software suite [29].

### 2.5. Microfluidic Measurement

**Figure 4:**
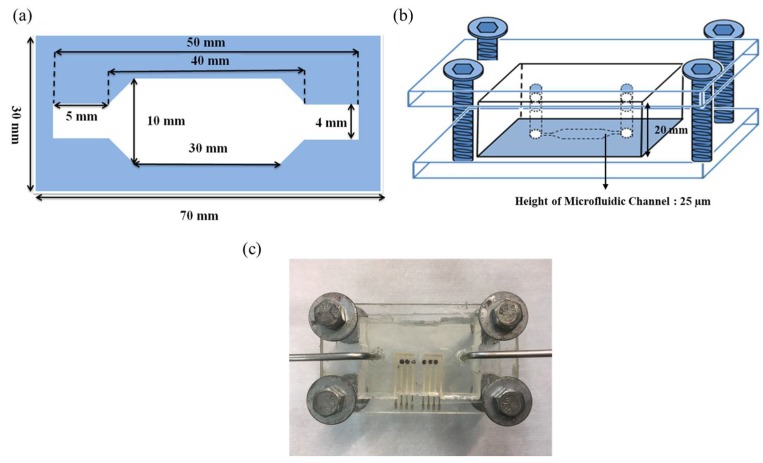
(**a**) The 2D and (**b**) 3D schematics of the microfluidic device. (**c**) The close-up image of the microfluidic device.

The microfluidic measurement system consists of a microchannel, a pump, an injector, a waste container, and a potentiometric measurement system. The test solution was injected into the device and flowed into the microchannel through the pump, which pushed it through the sensing area and controlled the flow rate. The 2D, 3D schematics, and the close-up image of the microfluidic device were shown in Figure 4. The height of the microfluidic channel is 25 µm, as shown in Figure 4b. The detailed information about the microfluidic measurement system can be found in our previous study [30]. In this work, the average sensitivities at different flow rates of 20 ml/h, 30 ml/h, 40 ml/h, and 50 ml/h were investigated.

## 3. Results and Discussion

### 3.1. Analysis of the Remote Monitoring for MBs-Urease/GO/TiO_2_ Urea Biosensor

**Figure 5:**
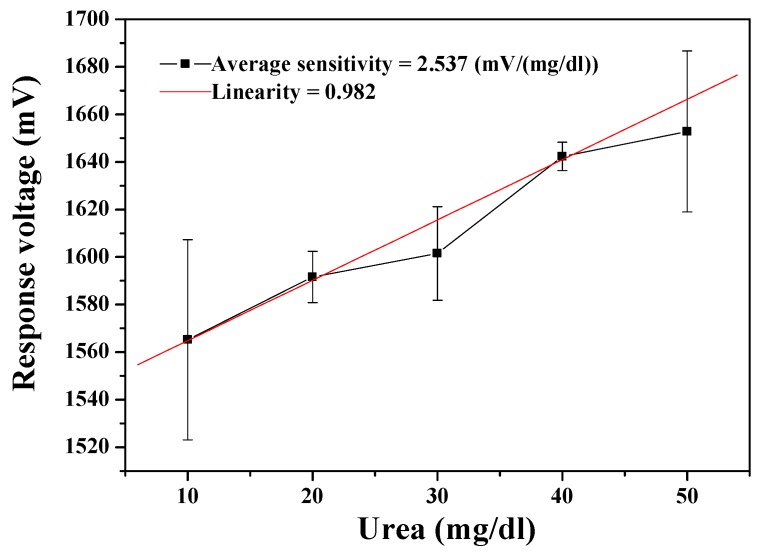
Responses of the urea biosensor based on MBs-urease/GO/TiO_2_ by integrating the wireless measurement system.

The urea biosensor based on MBs-urease/GO/TiO_2_ film was integrated with the wireless remote detection system. The results in Figure 5 show that the average sensitivity and linearity of the urea biosensor were 2.537 mV/(mg/dl) and 0.982, respectively. The dynamical measurement for ambient temperature was set at room temperature. Examining the wireless measurement results, the error bars are obtrusively large. This can be attributed to that fact that the noise resistance capability of the AD623 is low with its input resistance of about 2 GΩ. Additionally, the wireless system is based on the Arduino Mega 2560. When converting the analog to digital, the resolution of the Arduino Mega 2560 (10 bits) is not as high as the data acquisition card on the market, which is used for bioelectric signal in wired mode (16 bits). We consider it likely that this is the reason for the large observed error bars.

### 3.2. Analysis of the Remote Monitoring for MBs-Urease/GO/NiO Urea Biosensor

**Figure 6:**
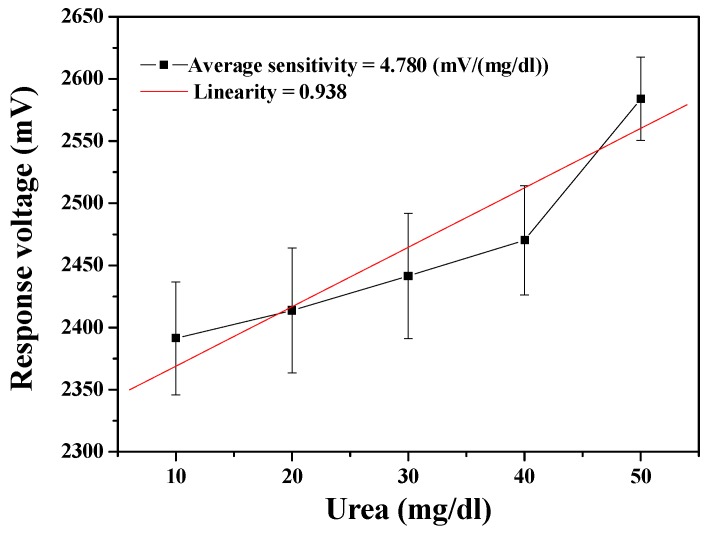
Responses of the urea biosensor based on MBs-urease/GO/NiO by integrating the wireless measurement system.

The performance of the MBs-urease/GO/NiO urea biosensor was measured through the wireless measurement system. As shown in Figure 6, the average sensitivity of the urea biosensor was 4.780 mV/(mg/dl) and with the linearity of 0.938. From the measured results, it can again be seen that the error bars are prohibitively large. The error bars in the measurement results can be caused by several possible factors in transmission through the wireless measurement system. When the device presumes that the electrochemical reaction is stable and continuous, the signal is amplified by the AD623 instrumentation amplifier. However, the noise may also be amplified at the same time as the signal, so that the data is mistakenly regarded as a signal. Further, the signal is converted from analog by the Arduino Mega 2560, with a limited signal resolution of 10 bits. That is, the resolution is too low to facilitate correct capture of the analog signal. The higher the sampling rate, the less easily the signal is distorted. When the sampling rate is higher (higher resolution), the signal is less likely to be distorted. In addition, the internal error rate of the signal converter must also be considered. When the signal is accepted by the XBee receiver, the received signal is then presented in LabVIEW. While all the aforementioned points must be addressed and corrected, the results in Figure 5 and Figure 6 exhibit the feasibility for remote detection of urea.

### 3.3. Sensing Properties of the MBs-Urease/GO/TiO_2_ Urea Biosensor with the Integrated Microfluidic Framework

**Figure 7:**
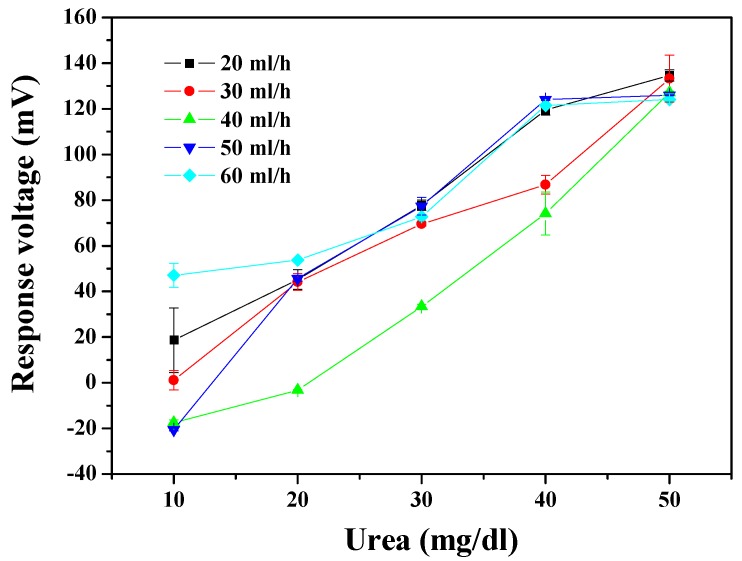
The responses of the urea biosensor based on MBs-urease/GO/TiO_2_ by integrating the microfluidic measurement system.

The dynamic measurement aspect of the urea biosensor was integrated with the microfluidic measurement system. The dynamic measured results are presented in Figure 7. The optimal sensing characteristics, of average sensitivity and linearity, were 4.256 mV/(mg/dl) and 0.995 at a flow rate of 40 ml/h. Average sensitivity and linearity of the urea biosensor are shown in Table 2; it specifies the sensing properties with different flow rates of 20 ml/h, 30 ml/h, 40 ml/h, 50 ml/h, and 60 ml/h. The result shows that the trend of sensitivity was unchanging as the flow rates gradually increased. When the flow rate was higher than 40 ml/h, the sensing property began to decay. This phenomenon may be attributed to the rapid flow rate causing the solution passing through the sensing area too quickly to achieve an equilibrium potential for the response voltage [20,31]. From a hydrodynamic point of view, fluids at a high flow rate are subject to flow-induced vibration (FIV) in the microfluidic level. Owing to the fluidic shock, vortex-induced vibrations (VIV) possibly are triggered by FIV [32]. From inferred reasons, it can be explained that the sensitivity was decreased at a high flow rate. Regardless of the reasons, the properties performed here, by the urea biosensor, reach the standard required for use under the dynamic conditions. This means that urea could be adequately detected under dynamic conditions by the proposed system.

### 3.4. Sensins Properties of the MBs-Urease/GO/NiO Urea Biosensor with the Integrated Microfluidic Framework

**Figure 8:**
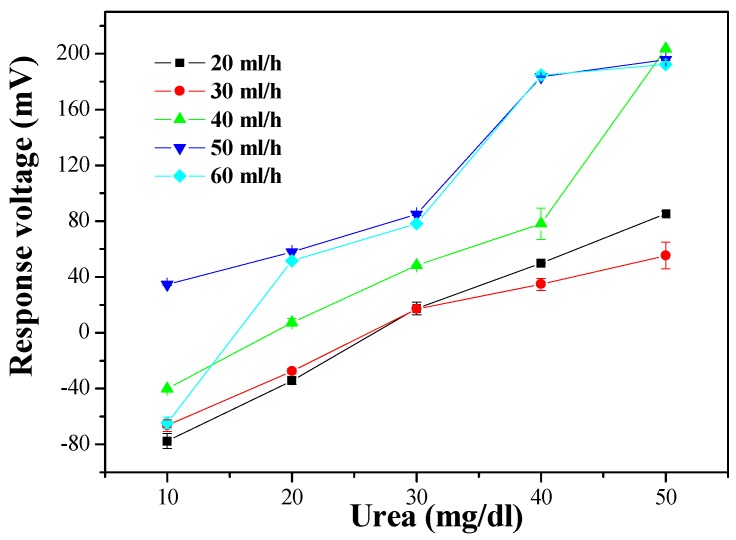
The responses of the urea biosensor based on MBs-urease/GO/NiO by integrating the microfluidic measurement system.

As shown in Figure 8, the optimal sensing properties, as measured by average sensitivity and linearity, were 5.582 mV/(mg/dl) and 0.959 at a flow rate of 40 ml/h. The measured average sensitivity and linearity of the urea biosensor at different flow rates are shown in Table 2. It can be seen that the biosensor decreased in sensitivity when the flow rate exceeded 40 ml/h, but the results still show that it maintained good sensitivity. Comparing the results of the MBs-urease/GO/TiO_2_ and MBs-urease/GO/NiO, it can be seen that the NiO-based matrix was more stable than the TiO_2_-based sensing matrix under dynamic detection. Under dynamic conditions, NiO promotes electrochemical reactions; this may be due to the high chemical stability of NiO [3,25,26].

## 4. Conclusions

The wireless monitoring for urea detection obtained a preliminary success, which the urea biosensors based on MBs-urease/GO/TiO_2_ and MBs-urease/GO/NiO integrated with Arduino Mega 2560. The observed results of the sensors based on MBs-urease/GO/TiO_2_ and MBs-urease/GO/NiO covered average sensitivity and linearity and were recorded as 2.537 mV/(mg/dl), 0.982, and 4.780 mV/(mg/dl), 0.938, respectively. In addition, the performances of the biosensors were performed under the dynamic conditions, with different flow rates of 20 ml/h, 30 ml/h, 40 ml/h, 50 ml/h and 60 ml/h. The results showed that of the NiO-based sensing matrix was more stable than the TiO_2_-based sensing matrix under dynamic detection because of the high chemical stability of NiO. The average sensitivity and linearity of the urea biosensor based on MBs-urease/GO/NiO were 5.582 mV/(mg/dl) and 0.959, respectively, at a flow rate of 40 ml/h.

In the future, in order to realize the remote and accurate detection of urea, the internal error rate of the wireless measurement system needs to be decreased, and the urea biosensors were applied to the measurement for real samples such as urine and serum. In addition, we will improve the volume of the microfluidic device and integrate it with the proposed urea biosensor achieving miniaturization. Further works are promising for remote urea detection under microfluidic flow and will be high precision.

## Figures and Tables

**Table 1:** Sputtering parameters for the different sensing matrices.

**Material**	TiO_2_	NiO
**Power (W)**	100	50
**Depostion Time (min)**	60	50
**Pressure (mTorr)**	30	3
**Gas flow (Ar:O_2_, sccm)**	20:1	10:0
**Ref.**	[25] 2019	[26] 2019

**Table 2:** The sensing properties of the different urea biosensors at different flow rates by integrating the microfluidic measurement system.

Flow Rate (ml/h)	MBs-Urease/GO/TiO_2_		MBs-Urease/GO/NiO	
	Average Sensitivity (mV/(mg/dl))	Linearity	Average Sensitivity (mV/(mg/dl))	Linearity
20	3.022	0.976	4.103	0.996
30	2.964	0.979	3.060	0.982
40	4.256	0.995	5.582	0.959
50	3.394	0.934	4.594	0.973
60	2.754	0.943	3.956	0.909
